# Altered Topological Organization of Cortical Network in Adolescent Girls with Idiopathic Scoliosis

**DOI:** 10.1371/journal.pone.0083767

**Published:** 2013-12-20

**Authors:** Defeng Wang, Lin Shi, Shangping Liu, Steve C. N. Hui, Yongjun Wang, Jack C. Y. Cheng, Winnie C. W. Chu

**Affiliations:** 1 Department of Imaging and Interventional Radiology, The Chinese University of Hong Kong, Shatin, New Territories, Hong Kong, China; 2 Research Center for Medical Image Computing, The Chinese University of Hong Kong, Shatin, New Territories, Hong Kong, China; 3 Shenzhen Research Institute, The Chinese University of Hong Kong, Shenzhen, China; 4 Department of Biomedical Engineering and Shun Hing Institute of Advanced Engineering, The Chinese University of Hong Kong, Shatin, New Territories, Hong Kong, China; 5 Department of Neurology, Beijing Tiantan Hospital, Capital Medical University, Beijing, China; 6 Department of Orthopaedics and Traumatology, The Chinese University of Hong Kong, Shatin, New Territories, Hong Kong, China; University of Maryland, College Park, United States of America

## Abstract

Adolescent idiopathic scoliosis (AIS) is a multifactorial disease affecting approximately 1–4% of teenagers especially girls at the age of 10–16, but its etiopathogenesis remains uncertain. Previous study has revealed that the cortical thickness in AIS patients is different from that in normal controls. Cortical thickness measurements are known to be strongly correlated between regions that are axonally connected. Hence, a hypothesis is proposed to study the possibility to demonstrate abnormal structural network revealed by cortical thickness in AIS patients. The aim of the study is to investigate abnormalities in the organization of the brain cortical network in AIS patients. This study included 42 girls with severe idiopathic scoliosis (14.7±1.3 years old) and 41 age-matched normal controls (NC, 14.6±1.4 years old). The brain cortex was partitioned into 154 cortical regions based on gyral and sulcal structure. The interregional connectivity was measured as the statistical correlations between the regional mean thicknesses across the subjects. We employed the graph theoretic analysis to examine the alteration in interregional correlation, small-world efficiency, hub distribution, and regional nodal characteristics in AIS patients. We demonstrated that the cortical network of AIS patients fully preserved the small-world architecture and organization, and further verified the hemispheric asymmetry of AIS brain. Our results indicated increased central role of temporal and occipital cortex and decreased central role of limbic cortex in AIS patients compared with controls. Furthermore, decreased structural connectivity between hemispheres and increased connectivity in several cortical regions were observed. The findings of the study reveal the pattern of structural network alteration in AIS brain, and would help in understanding the mechanism and etiopathogenesis of AIS.

## Introduction

Idiopathic scoliosis is a 3-dimensional spinal deformity that occurs most commonly in adolescent girls and affects about 1–4% of school teenagers worldwide. A more distinct indication of AIS is the prominence of back when they fully bend forward. Current studies indicate that the etiopathogenesis of AIS remains unknown and scientists believe that it is a multifactorial disease which involves intrinsic factors such as the growth of vertebral body, abnormal morphometry of the posterior elements, asymmetrical growth of the neurocentral cartilage etc., and extrinsic factors such as genetic components and chromosomes, extra-spinal left-right asymmetry, body growth and development, imbalance of muscle, dysfunction of the nervous system, somatosensory function, vestibular system, length of spinal cord, abnormal platelets and abnormality in melatonin metabolism [1]. Central nervous system is one of the major factors related to the etiopathogenesis of AIS.

Recently, various neuroanatomical studies have been proposed to explore the etiopathogenesis of AIS patients. Structural brain MRI analyses, such as region volume [2], cortical thickness [3], comprehensive morphometry of corpus callosum [4], vestibular morphoanatomy [5], volume-based morphometry [6], have been used to investigate the morphological change in the brains of AIS patients. The preliminary analysis of regional brain volume has indicated the anatomical asymmetry in brain regions functionally related to somatic motorcontrol and coordination [2]. The morphoanatomical changes of vestibular system and vestibular network were found to be associated with subclinical postural, vestibular, and proprioceptive dysfunctions in AIS [3], [5]. We have recently reported the thinning pattern of cerebral cortex in AIS patients. The findings of cortical thinning pattern also lead us to make a hypothesis: is it possible that altered organization pattern of large-scale structural network could be revealed in AIS patients compared with age-matched healthy individuals?

The small-world architecture (popularly known as six degrees of separation) first quantified by Watts and Strogatz [7] has been widespread utilized to describe the topological properties of networks in social, biological, and man-made systems. Recently the terms of “network” and “small-worldness” began to raise more interest in the aspect of brain organization which was demonstrated to share similar properties with real-world networks of different nature (e.g., social networks, the Internet, et al) [8], [9]. Specifically, when the human brain is considered to be a complex network with nodes representing brain regions and edges representing inter-regional interactions, brain network properties can be derived from generated graphs [9]. On the other hand, it has been confirmed that the structural features of human cortex convey brain connectivity information, e.g., the cortical thickness are known to be strongly correlated between regions that are axonally connected like Broca's and Wernicke's Areas [10]. Thus, a whole-brain cortical network can be obtained from structural MRI data based on a matrix of correlations in cortical thickness between all pairs of parcellated regions [11], [12]. Indeed, previous study has demonstrated that the variations in human regional cortical thickness follow the small-world topology at the macroscale level [12], and the alteration in topological organization of brain cortical network has been demonstrated useful to investigate the brain development [13], age- and gender-related change [14], [15], and the characteristic mechanisms of a series of neurodegenerative diseases [16], [17]. Our goal in this study is to investigate the abnormalities of the organization of the brain cortical network in AIS patients by applying cortical thickness measurements combined with the graph theoretical analysis.

## Materials and Methods

### Subjects

This study included 42 girls with severe idiopathic scoliosis (14.7±1.3 years old, range 13–18) and 41 age-matched normal controls (NC, 14.6±1.4 years old, range 12–18), which were recruited from the Prince of Wales Hospital in Hong Kong. Inclusion criteria for AIS patients included: (1) 12≤ age ≤18 years; (2) right thoracic curves; (3) Cobb angle ≥40°; (4) availability of collection the patient's medical history from the caregiver. The AIS patients had an average Cobb angle of 54.5° (range 40–90°). Exclusion criteria for both AIS patients and NC included any history of head injury, headache, back injury, weakness or numbness in any limbs, urinary incontinence, nocturnal enuresis or any space occupying lesion found on screening MR imaging. There were no significant differences in age and education between the two groups (all *P*>0.05). All the subjects were right-handed. All the subjects were required to be normal in detailed neurological examinations. Ethical approval was obtained from the university and hospital ethics committee. Informed written consent was obtained from each subject and her parents.

### MRI acquisition

All subjects were scanned using a clinical 1.5T MRI scanner (Sonata, Siemens, Erlanger, Germany) with a quadrature head coil. For each subject, high resolution T1-weighted images covering the whole brain were acquired in the axial orientation using magnetization prepared rapid acquisition gradient echo (MPRAGE) with the following parameters: repetition time  = 2070 ms, echo time  = 3.93 ms, inversion time  = 1110 ms, field of view  = 230 mm, flip angle  = 15°, matrix  = 256×256, slice  = 192, voxel size  = 0.9×0.9×0.9 mm^3^.

### Cortical thickness mapping

**Figure 1:**
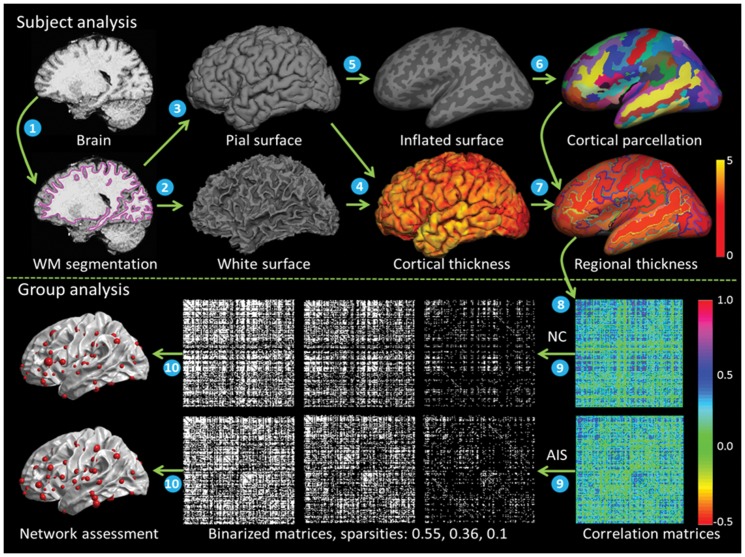
Whole schematic flowchart of cortical structural network construction. (1) white matter (WM) segmentation, (2) and (3) white surface and pial surface generation, (4) cortical thickness measurement, (5) surface Inflation, (6) cortical parcellation, (7) regional thickness calculation, (8) interregional correlation across subjects, (9) correlation matrices binarization, (10) network assessment.

The whole schematic flowchart of cortical structural network construction is shown in Figure 1. For each subject, freesurfer toolkit (http://surfer.nmr.mgh.harvard.edu) was used to analyze the anatomical MPRAGE images. The freesurfer procedure involving white matter segmentation [18], subcortical mass labeling and filling [19], tessellation and topology fixing, topological defects correction [20], white and pial cortical surface generation [21], and cortical thickness measurement [21]. The cortical thickness at each vertex was measured as the shortest distance to the white surface from each point on the pial surface averaged with the shortest distance to the pial surface from each point on the white surface.

### Regional thickness calculation

Using spherical registration [22], the individual inflated surface was normalized to a spherical template, and the brain cortexes were partitioned into 154 cortical regions base on a spherical atlas [23]. The cortical thickness for each cortical region was measured as the average thickness of all vertices defined as belonging to that region.

### Interregional correlation

The structural connectivity between two cortical regions was defined as the statistical correlation between the regional thicknesses across subjects. Before the correlation computing, the linear regression was performed to remove the effects of age and mean cortical thickness on the full set of regional thickness in each region. The residuals of the regression were then substituted for the raw regional thickness values. The interregional correlation matrix (154×154) of both NC and AIS group was generated by calculating the Pearson correlation coefficients across NC and AIS subjects between the residual thicknesses of each pair of cortical regions.

### Threshold selection

The cortical network is represented by a binarized matrix. Since currently no accepted strategy to define an optimal threshold, the correlation matrix was usually thresholded repeatedly over a series of thresholds. If the same correlation threshold was applied to the correlation matrices of both AIS and NC groups, the number of nodes and edges in two cortical networks would differ from each other and the group difference would not be purely reflected by the alterations of network properties. To control this effect, a range of sparsites were used to substitute the correlation thresholds to binarize the matrices. Sparsity is defined as the total number of edges divided by the maximum possible number of edges in a network, which would result two networks of both groups with same number of edges or wiring cost [24].

### Small-world efficiency

The network efficiency metrics, first proposed by Watts and Strogatz [7], were used to reflect the efficiency in communication and organization over a network system. Specifically, the small-world efficiency introduced here was a quantitative analysis on how efficiently the network of human brain exchanges information at the global and local levels [25]. The global efficiency of a network graph *G* was defined as the inverse of the harmonic mean of the shortest path length between every two nodes, 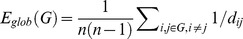, where 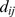 is the length of the shortest path connecting node *i* and *j*, and *n* is the total number of nodes. The local efficiency of a network graph *G* was defined as the average of the local efficiencies of each node in a network, 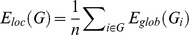, where 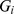 is the subgraph composed of the nearest neighbors of node *i*. The cortical network can be considered to be a small-world network if it meets the criteria 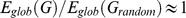, 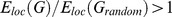 [25], where the 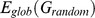 and 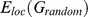 are the averages of global efficiencies and efficiencies of 100 matched random networks that preserve the same number of nodes, edges, and degree distribution as the real network.

### Regional nodal characteristics

The betweenness centrality was essentially a measurement of the influence of a node over the information flow between itself and other nodes. The betweenness centrality 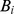 of each node *i* was defined as the fraction of all shortest paths in the network that pass through it [26], 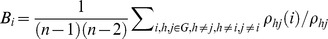, where 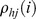 is the number of shortest path between nodes *h* and *j* that pass through node *i*, *n* is the total number of nodes. The mean betweenness centrality 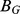 was calculated as the average of the values of the nodal betweenness over all nodes. The node betweenness was normalized by the mean betweenness centrality 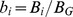.

The hubs of the cortical networks were defined as the regions with high values of normalized betweenness (at least one standard deviation greater than the average normalized betweenness). As was headed up in the introduction, the nodes represented different brain regions. Then the hubs are considered to be the brain regions playing significant roles in information transferring and integrating in whole brain communication [11].

The regional efficiency 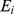 of each node *i* was defined as the inverse of harmonic mean of the shortest path length between itself and all other nodes [24] 
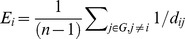
. The regional efficiency measures the average shortest path length between a given node and all the other nodes in the network, which is used to quantify the importance of each node in the communication within the network.

The vulnerability 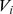 of each node *i* was defined as the drop in global efficiency when this node and its connections were removed from the network graph, reflecting [27] 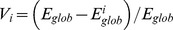, where 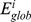 is the global efficiency of the network after removing node *i*. The global vulnerability 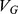 of the entire network was defined as the maximum vulnerability for all of its nodes. The vulnerability analysis is a way to identify critical components and can quantitatively measure the damage on the network performance caused by the hypothetical failure of its elements.

### Statistical Analysis

#### Interregional correlation

Differences in correlation of regional thickness between the AIS and NC groups were examined using z-test. Prior to the statistic, the Pearson correlation coefficients were converted into normally distributed z values using Fisher's r-to-z transform. The between-group differences in correlations were determined by comparing these z values using z-test [28]. The false discovery rate (FDR) [29] with *q*  = 0.05 was used to correct the multiple comparisons.

#### Network characteristics

Differences in overall graph characteristics (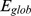, 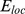) and regional nodal characteristics (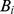, 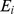) between the AIS and NC groups were examined using nonparametric permutation test [30]. The permutation test proceeded as follows. First, the network characteristics were calculated separately at a given sparsity for each group and their differences were obtained. Then the subjects in both groups were pooled and randomly assigned to either one of two groups consisting of the same size as the original AIS and NC groups. The correlation matrices for these two new groups were recalculated and binarized using the same threshold as in the real network. The network characteristics were calculated for each random group and their differences were obtained. This procedure was repeated for 1,000 times. The 95 percentile points of each distribution were used as the critical values for a one-tailed testing the overall graph characteristics (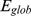, 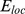, 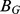, 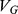) of type I error of 0.05. The 99 percentile points of each distribution were used as the critical values for a one-tailed test of the regional nodal characteristics (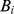, 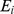) of type I error of 0.01.

## Results

### Change in interregional correlation

**Figure 2:**
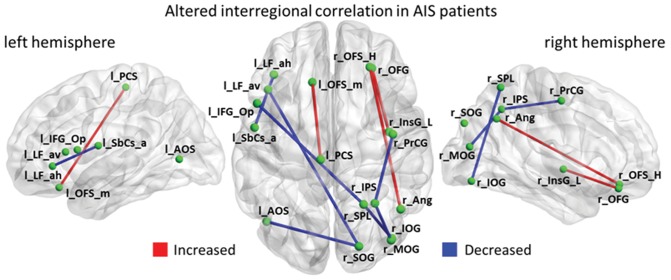
Altered interregional correlations in AIS patients. The red and blue lines indicate significantly increased and decreased correlations between pairs of regions respectively.

The altered interregional correlations in AIS patients were shown in Figure 2. Increased correlation between the left olfactory sulcus [l_OFS_m] and the left paracentral sulcus [l_PCS] (*P* = 0.00005^★^, ^★^ indicates the correlation survived critical FDR threshold), between the right orbital sulci [r_OFS_H] and the right angular gyrus [r_Ang] (*P* = 0.00007^★^), and between the right orbital gyri [r_OFG] and the right long insular gyrus [r_InsG_L] (*P* = 0.0003^★^), were observed in the AIS patients compared with the controls. Decreased correlation between the left anterior horizontal limb of lateral sulcus [l_LF_ah] and the left anterior subcentral sulcus [l_SbCs_a] (*P* = 0.00023^★^), between the right middle occipital gyrus [r_MOG] and the right intraparietal sulcus and transverse parietal sulci [r_IPS] (*P* = 0.00026^★^), between the right inferior occipital gyrus and the sulcus [r_IOG] and the right superior parietal lobule [r_SPL] (*P* = 0.00023^★^), between the right intraparietal sulcus and the transverse parietal sulci [r_IPS] and the right precentral gyrus [r_PrCG] (*P* = 0.00025^★^), between the left anterior occipital sulcus [l_AOS] and the right superior occipital gyrus [r_SOG] (*p* = 0.0002^★^), between the left anterior ascending limb of lateral sulcus [l_LF_av] and the right superior occipital gyrus [r_SOG] (*P* = 0.00022^★^), and between the left opercular part of inferior frontal gyrus [l_IFG_Op] and the right superior parietal lobule [r_SPL] (*P* = 0.00018^★^) were observed in the AIS patients compared with the controls.

### Small-world efficiency

**Figure 3:**
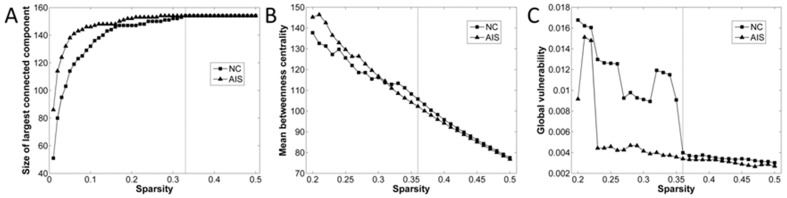
Sparsity thresholds used for cortical networks construction. (A) The size of the largest connected component of structural networks as function of sparsity, (B) The mean betweenness centrality as function of sparsity, (C) The global vulnerability as function of sparsity.

To investigate the network characteristics, the cortical networks were constructed using a series of sparsities thresholds. A specific sparsity 0.36 was determined by mean betweenness centrality 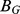 and global vulnerability 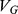 to ensure that all cortical regions were included in the cortical networks while keeping the network more stable (Figure 3). A range of sparsities thresholds (0.36≤ sparsity ≤0.5, step  = 0.01) were used to verify the network efficiency of cortical networks. Both AIS and NC groups exhibit efficient small-world topology (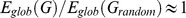, 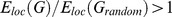) in cortical networks, and no significant difference in global efficiency and local efficiency (0.36≤ sparsity ≤0.5, all *P*>0.05, uncorrected) was found.

### Distribution of hub regions

**Figure 4:**
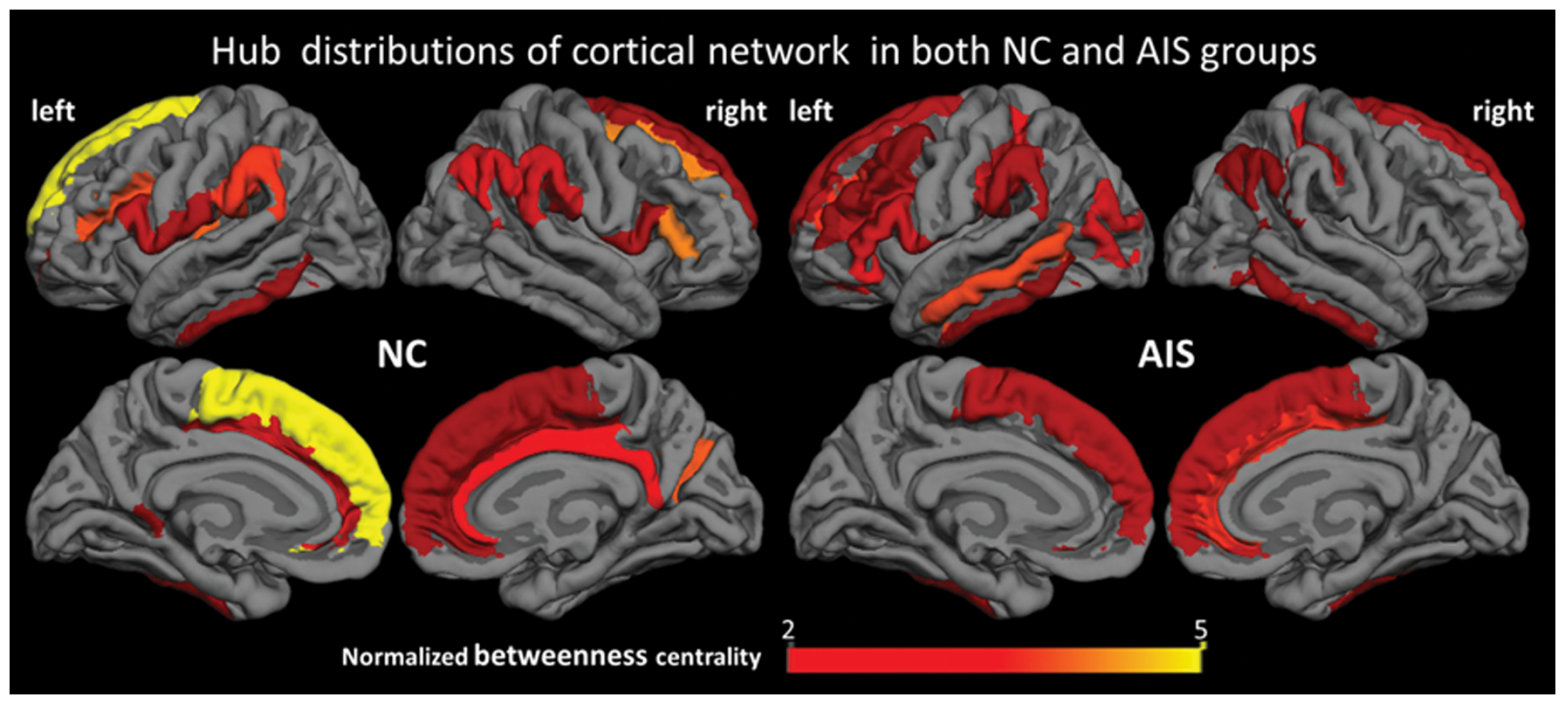
Hub distributions and node-specific normalized betweenness of cortical networks in both NC and AIS groups. Hubs in the NC group are distributed in 12 left hemispherical regions, 10 right hemispherical regions (9 frontal regions, 1 temporal region, 5 parietal regions, 1 occipital region, 4 limbic regions, 1 parieto-frontal region, and 1 insular region). Hubs in the AIS group are distributed in 17 left hemispherical regions, 9 right hemispherical regions (10 frontal regions, 5 temporal regions, 4 parietal regions, 3 occipital regions, 1 limbic region, 1 parieto_frontal region, 1 occipito-temporal region, and 1 insular region).

In this study, 22 and 26 hubs were identified in cortical network of NC and AIS groups respectively, which were thresholded by a specific sparsity threshold 0.36 (Figure 4). Hubs in the NC group were distributed in 12 left hemispherical regions and 10 right hemispherical regions (9 frontal regions, 1 temporal region, 5 parietal regions, 1 occipital region, 4 limbic regions, 1 parieto-frontal region, and 1 insular region). Hubs in the AIS group were distributed in 17 left hemispherical regions and 9 right hemispherical regions (10 frontal regions, 5 temporal regions, 4 parietal regions, 3 occipital regions, 1 limbic region, 1 parieto-frontal region, 1 occipito-temporal region, and 1 insular region). Among them, 10 hubs were the same for both groups including the bilateral middle frontal gyrus, the opercular part of inferior frontal gyrus, the superior frontal gyrus, the middle occipital gyrus, the supramarginal gyrus, the inferior temporal gyrus, and the inferior frontal sulcus in left hemisphere, the angular gyrus, and the cingulate sulcus in right hemisphere. Twelve cortical regions including the isthmus of cingulate gyrus, the long gyrus of insula, the subcentral sulci, the cingulate sulcus, and the frontomarginal sulcus in left hemisphere, and the cingulate gyrus, the opercular and triangular part of inferior frontal gyrus, the supramarginal gyrus, the superior frontal gyrus, the sulcus intermedius primus, and the parieto-occipital sulcus in right hemisphere, were identified as hubs in the NC group but not in the AIS group. Sixteen cortical regions, the bilateral middle frontal sulcus, the bilateral postcentral sulcus, the bilateral superior temporal sulcus, the orbital and triangular part of inferior frontal gyrus, the middle temporal gyrus, the inferior segment of the circular sulcus of insula, the orbital sulcus, and the paracentral sulcus in left hemisphere, the inferior temporal gyrus, the calcarine sulcus, and the occipito-temporal sulcus in right hemisphere, were identified as hubs in the AIS group but not in the NC group.

### Change in nodal characteristics

**Figure 5:**
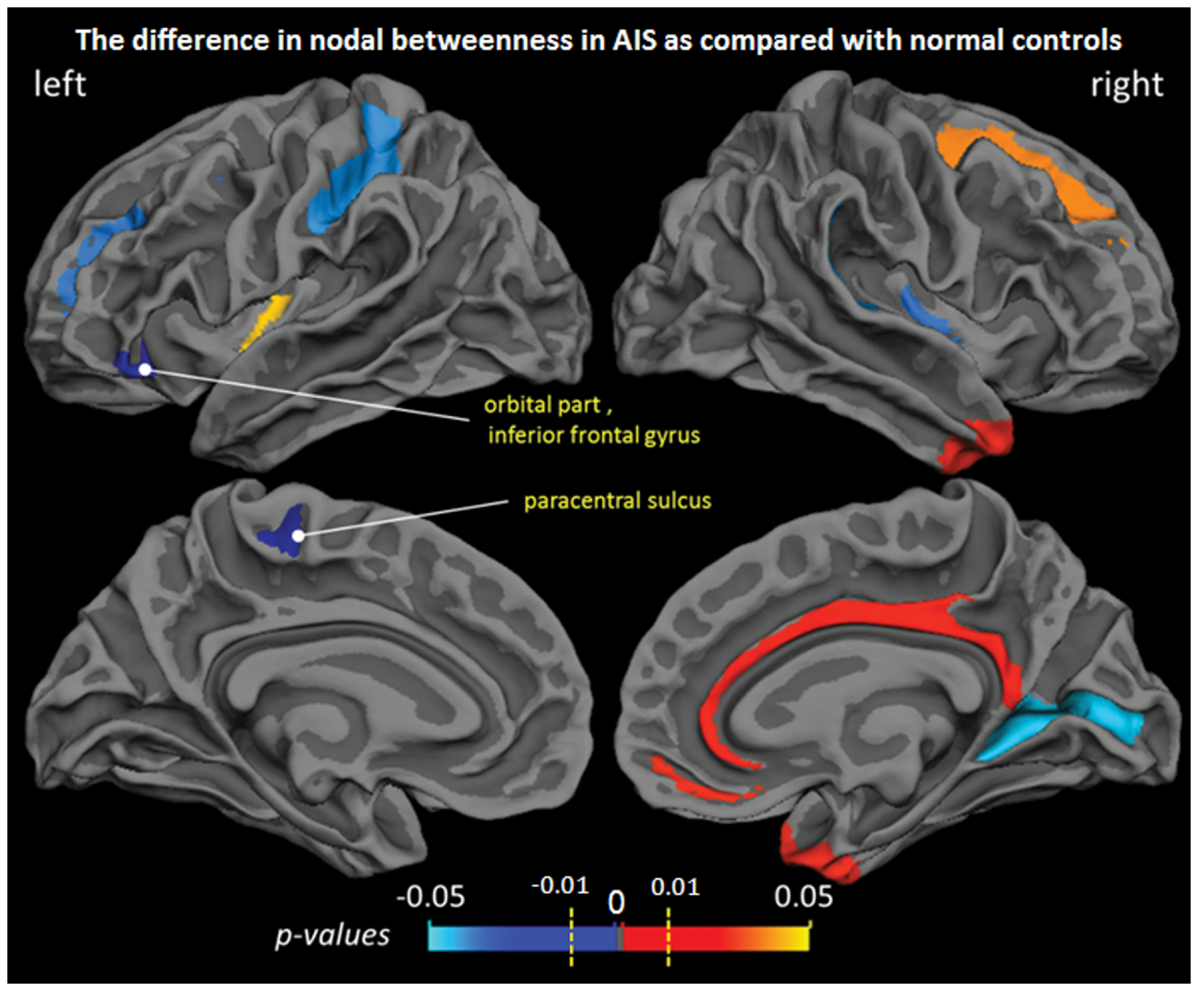
Group differences of node-specific betweenness centrality (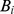) between NC and AIS groups. The mean *p*-values of a range of sparsities from 0.36 to 0.5 were overlaid on the cortical surface. Significant increase was observed in orbital part of left inferior frontal gyrus and left paracentral sulcus.

**Figure 6:**
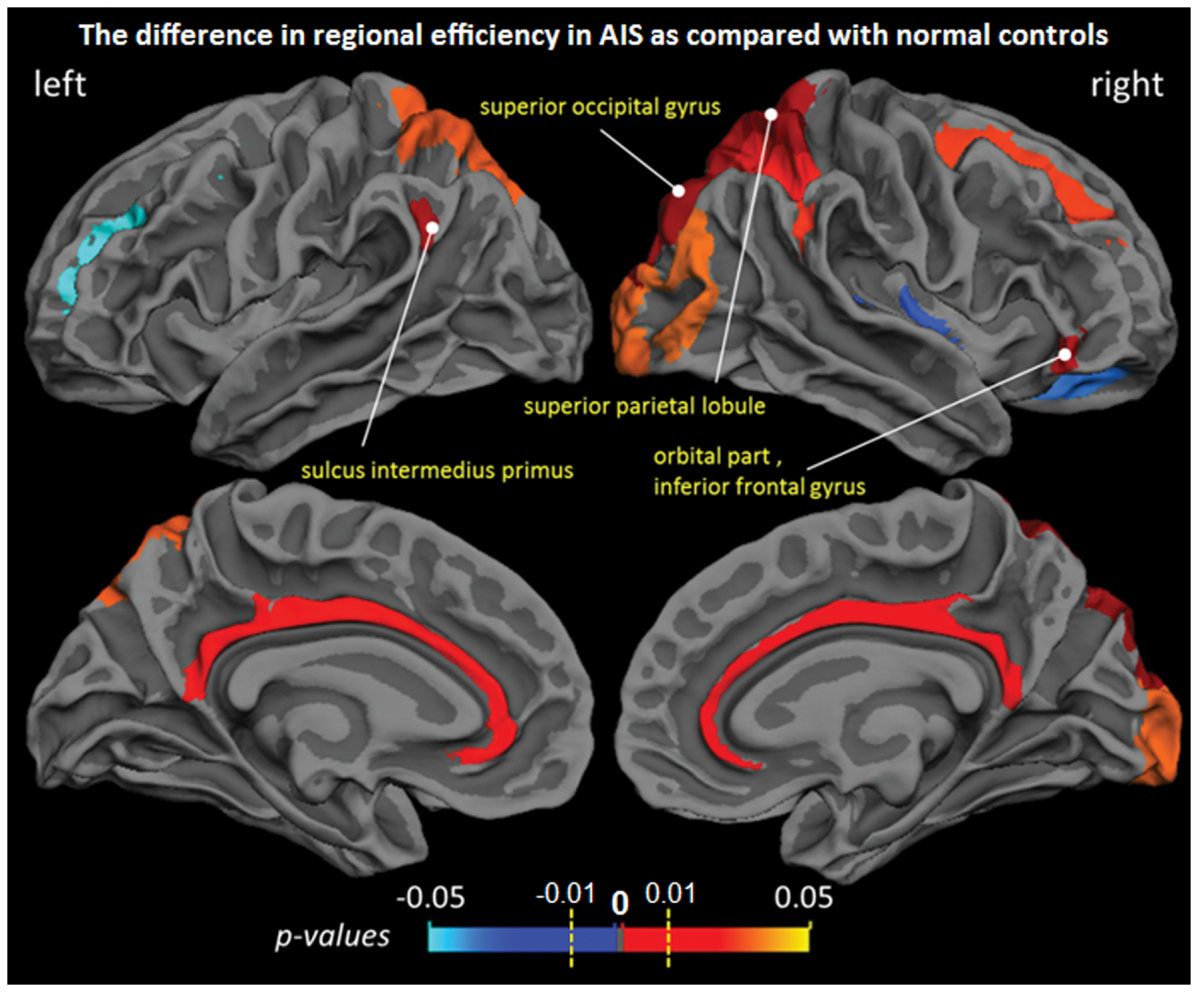
Group differences of regional efficiency (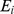) between NC and AIS groups. The mean *p*-values of a range of sparsities from 0.36 to 0.5 were overlaid on the cortical surface. Significant increase was observed in left sulcus intermedius primus, right superior occipital, right superior parietal lobule, and orbital part of right inferior frontal gyrus.

A range of sparsities thresholds (0.36≤ sparsity ≤0.5, step  = 0.01) were also used to investigate the nodal characteristics of cortical networks. The between-group differences of nodal characteristics (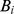, 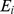) were shown in Figure 5 and 6, where the mean *p*-values of the series of sparsities were overlaid on the mean cortical surface. Significantly decreased betweenness centrality 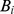 in AIS group was found in the orbital part of left inferior frontal gyrus (*P*<0.01, when 0.38≤ sparsity ≤0.48, uncorrected, 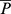  = 0.0081, 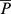 indicates the mean *p*-values of the series of sparsities from 0.36 to 0.5) and the left paracentral sulcus (*P*<0.01, when 0.36≤ sparsity ≤0.5, uncorrected, 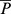  = 0.0048) (Figure 5). Significantly increased regional efficiency 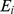 in AIS group was found in the left sulcus intermedius primus (*P*<0.01, when 0.36≤ sparsity ≤0.5, uncorrected, 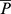  = 0.0054), the right superior occipital gyrus (*P*<0.01, when 0.36≤ sparsity ≤0.5, uncorrected, 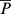  = 0.0023), the right superior parietal lobule (*P*<0.01, when 0.37≤ sparsity ≤0.49, uncorrected, 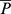  = 0.0059), and the orbital part of right inferior frontal gyrus (*P*<0.01, when 0.36≤ sparsity ≤0.37 and 0.43≤ sparsity ≤0.5, uncorrected, 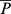  = 0.0085) (Figure 6).

## Discussion

### Altered interregional correlation

Interregional correlation which is a statistical association reflects the structural connectivity or connection between pairs of cortical regions. Only decreased between-hemisphere correlations were observed, which demonstrated that the AIS only negatively affects the between-hemisphere connectivity. Three orbitofrontal regions with increased correlations were observed in AIS patients. Particularly, since the insular cortex is in a position to convey diverse sensory information to the orbitofrontal cortex [31], the increased connectivity of right orbitofrontal-insular circuit in AIS patients may relate with the right thoracic convexity. The reduced correlation in left anterior ascending and horizontal limb of lateral sulcus may be caused by decreased cortical thickness in left lateral sulcus (sylvian fissure) [3]. Most of brain regions with altered correlation were included in the vestibular cortical network previously revealed by caloric stimulation and functional MRI [32], and these findings were also supported by the previous finding of vestibular dysfunction [33] and abnormal morphoanatomy [5] in AIS patients.

### Small-world efficiency

Both AIS and NC subjects were found to demonstrate small-world architectures and exhibit both high global and local efficiency, which were compatible with previous structural brain networks studies [11], [14]–[16]. The comparison of global and local efficiency between patients and controls in the current study revealed unchanged network organization in AIS over a wide range of sparsities. Our finding suggests that the small-world topological characteristics were fully preserved in AIS patients.

### Alteration of Hub Distribution

The identified hubs in this study were consistent with those obtained from previous similar studies [15]–[17]. There was no significant difference of the hub distribution in frontal (NC/AIS: 9/10), parietal (NC/AIS: 5/4), and parieto-frontal (NC/AIS: 1/1) cortex between NC and AIS groups. In this study, most identified hubs of both groups were frontal and parietal regions, which are in line with the previous studies on brain graphs [34]. Nevertheless, the number of hubs in temporal cortex of AIS group was increased from 1 to 5 compared with NC. This may be related with the dysfunction and abnormal morphoanatomy of vestibular system in the temporal lobe observed in AIS patients [5], [33] and supported by the fact that AIS patients are with thinner cortex in bilateral lateral sulcus (sylvian fissure) [3]. Possibly effected by the decreased cortical thickness in lateral occipital regions [3], the number of hubs in the occipital cortex was also increased (from 1 to 3). Interestingly, the number of hubs in the limbic cortex of AIS group was reduced from 4 to 1 compared with NC, which may be a possible compensatory mechanism for increased hub number of temporal and occipital cortex. In short, increased central role of temporal and occipital cortex and decreased central role of limbic cortex were observed in AIS patients. Furthermore, the altered distribution pattern of hubs in left (NC/AIS: 12/17) and right hemisphere (NC/AIS: 10/9) indicated the hemispheric asymmetry in AIS patients, and this is in line with the previous studies of AIS [3], [35], [36]. In this study, all AIS patients were with right thoracic curves, supporting the findings of hemispheric asymmetry of hub distribution.

### Altered Nodal Characteristics

Reduced betweenness was observed in the left paracentral sulcus, which belongs to the primary motor area being responsible for generating neural impulses that pass down to the spinal cord and control the execution of movement. The change suggests that the left paracentral sulcus of AIS patient has decreased influence in the brain communication compared with that of NC. Enhanced regional efficiency was observed in the right superior parietal gyrus, which is a part of the posterior parietal cortex. The posterior parietal cortex is also considered to be part of motor cortical area. The change suggests the increased connectivity of the right superior parietal to all other nodes in AIS patient compared with that in NC. Although no significant abnormal activation was observed among these currently identified regions in previous functional study [37], our findings may also suggests an altered pattern of structural connectivity of motor-related cortex in AIS. Furthermore, the results, 1) unchanged global and local efficiency, 2) four regions with enhanced regional efficiency, and 3) no region with reduced regional efficiency, demonstrates that the enhanced ability of commutation only occurred in several cortical regions.

## Conclusions

We applied cortical thickness measurement, interregional correlation, and graph theory to analyze the topological organization cortical network in AIS patients. This study demonstrates for the first time that the AIS brain was associated with changed between-hemisphere connectivity, and changed ability of communication of brain cortical networks. Our results indicate that the cortical network of AIS patients fully preserves the small-world architecture and organization, and further verify the hemispheric asymmetry of AIS brain. These findings were compatible with and complemented our previous neuroanatomical findings, thus enhancing the understanding of underlying etiology of AIS.

## References

[1] Kouwenhoven JW, Castelein RM (2008) The pathogenesis of adolescent idiopathic scoliosis: review of the literature. Spine (Phila Pa 1976) 33: 2898–2908. 19092622 10.1097/BRS.0b013e3181891751

[2] Liu T, Chu WC, Young G, Li K, Yeung BH, et al (2008) MR analysis of regional brain volume in adolescent idiopathic scoliosis: neurological manifestation of a systemic disease. J Magn Reson Imaging 27: 732–736. 18302230 10.1002/jmri.21321 PMC2430659

[3] Wang D, Shi L, Chu WC, Burwell RG, Cheng JC, et al (2012) Abnormal cerebral cortical thinning pattern in adolescent girls with idiopathic scoliosis. Neuroimage 59: 935–942. 21872666 10.1016/j.neuroimage.2011.07.097

[4] Wang D, Shi L, Chu WC, Paus T, Cheng JC, et al (2009) A comparison of morphometric techniques for studying the shape of the corpus callosum in adolescent idiopathic scoliosis. Neuroimage 45: 738–748. 19280700 10.1016/j.neuroimage.2008.12.068

[5] Shi L, Wang D, Chu WC, Burwell GR, Wong TT, et al (2011) Automatic MRI segmentation and morphoanatomy analysis of the vestibular system in adolescent idiopathic scoliosis. Neuroimage 54 Suppl 1S180–S188. 20382235 10.1016/j.neuroimage.2010.04.002

[6] Shi L, Wang D, Chu WC, Burwell RG, Freeman BJ, et al (2009) Volume-based morphometry of brain MR images in adolescent idiopathic scoliosis and healthy control subjects. AJNR Am J Neuroradiol 30: 1302–1307. 19386729 10.3174/ajnr.A1577 PMC7051582

[7] Watts DJ, Strogatz SH (1998) Collective dynamics of ‘small-world’ networks. Nature 393: 440–442. 9623998 10.1038/30918

[8] Voges N, Aertsen A, Rotter S (2012) Structural models of cortical networks with long-range connectivity. Math Probl Eng 2012 :

[9] Sporns O (2011) The human connectome: a complex network. Ann N Y Acad Sci 1224: 109–125. 21251014 10.1111/j.1749-6632.2010.05888.x

[10] Lerch JP, Worsley K, Shaw WP, Greenstein DK, Lenroot RK, et al (2006) Mapping anatomical correlations across cerebral cortex (MACACC) using cortical thickness from MRI. Neuroimage 31: 993–1003. 16624590 10.1016/j.neuroimage.2006.01.042

[11] Bassett DS, Bullmore E, Verchinski BA, Mattay VS, Weinberger DR, et al (2008) Hierarchical organization of human cortical networks in health and schizophrenia. J Neurosci 28: 9239–9248. 18784304 10.1523/JNEUROSCI.1929-08.2008 PMC2878961

[12] He Y, Chen ZJ, Evans AC (2007) Small-world anatomical networks in the human brain revealed by cortical thickness from MRI. Cereb Cortex 17: 2407–2419. 17204824 10.1093/cercor/bhl149

[13] Fan Y, Shi F, Smith JK, Lin W, Gilmore JH, et al (2011) Brain anatomical networks in early human brain development. Neuroimage 54: 1862–1871. 20650319 10.1016/j.neuroimage.2010.07.025 PMC3023885

[14] Gong G, Rosa-Neto P, Carbonell F, Chen ZJ, He Y, et al (2009) Age- and gender-related differences in the cortical anatomical network. J Neurosci 29: 15684–15693. 20016083 10.1523/JNEUROSCI.2308-09.2009 PMC2831804

[15] Wu K, Taki Y, Sato K, Kinomura S, Goto R, et al (2012) Age-related changes in topological organization of structural brain networks in healthy individuals. Hum Brain Mapp 33: 552–568. 21391279 10.1002/hbm.21232 PMC6870030

[16] He Y, Chen Z, Evans A (2008) Structural insights into aberrant topological patterns of large-scale cortical networks in Alzheimer's disease. J Neurosci 28: 4756–4766. 18448652 10.1523/JNEUROSCI.0141-08.2008 PMC6670444

[17] Yao Z, Zhang Y, Lin L, Zhou Y, Xu C, et al (2010) Abnormal cortical networks in mild cognitive impairment and Alzheimer's disease. PLoS Comput Biol 6: e1001006. 21124954 10.1371/journal.pcbi.1001006 PMC2987916

[18] Dale AM, Fischl B, Sereno MI (1999) Cortical surface-based analysis. I. Segmentation and surface reconstruction. Neuroimage 9: 179–194. 9931268 10.1006/nimg.1998.0395

[19] Fischl B, Salat DH, Busa E, Albert M, Dieterich M, et al (2002) Whole brain segmentation: automated labeling of neuroanatomical structures in the human brain. Neuron 33: 341–355. 11832223 10.1016/s0896-6273(02)00569-x

[20] Fischl B, Liu A, Dale AM (2001) Automated manifold surgery: constructing geometrically accurate and topologically correct models of the human cerebral cortex. IEEE Trans Med Imaging 20: 70–80. 11293693 10.1109/42.906426

[21] Fischl B, Dale AM (2000) Measuring the thickness of the human cerebral cortex from magnetic resonance images. Proc Natl Acad Sci U S A 97: 11050–11055. 10984517 10.1073/pnas.200033797 PMC27146

[22] Fischl B, Sereno MI, Dale AM (1999) Cortical surface-based analysis. II: Inflation, flattening, and a surface-based coordinate system. Neuroimage 9: 195–207. 9931269 10.1006/nimg.1998.0396

[23] Fischl B, van der Kouwe A, Destrieux C, Halgren E, Segonne F, et al (2004) Automatically parcellating the human cerebral cortex. Cereb Cortex 14: 11–22. 14654453 10.1093/cercor/bhg087

[24] Achard S, Bullmore E (2007) Efficiency and cost of economical brain functional networks. PLoS Comput Biol 3: e17. 17274684 10.1371/journal.pcbi.0030017 PMC1794324

[25] Latora V, Marchiori M (2001) Efficient behavior of small-world networks. Phys Rev Lett 87: 198701. 11690461 10.1103/PhysRevLett.87.198701

[26] Freeman LC (1977) A set of measures of centrality based on betweenness. Sociometry 40: 35–41.

[27] Costa L, Rodrigues FA, Travieso G, Villas Boas PR (2007) Characterization of complex networks: A survey of measurements. Adv Phys 56: 167–242.

[28] Cohen J, Cohen P (1983) Applied multiple regression/correlation analysis for the behavioral sciences. Hillsdale, NJ: Erlbaum

[29] Benjamini Y, Hochberg Y (1995) Controlling the false discovery rate - a practical and powerful approach to multiple testing. J R Stat Soc Series B Stat Methodol 57: 289–300.

[30] Bullmore ET, Suckling J, Overmeyer S, Rabe-Hesketh S, Taylor E, et al (1999) Global, voxel, and cluster tests, by theory and permutation, for a difference between two groups of structural MR images of the brain. IEEE Trans Med Imaging 18: 32–42. 10193695 10.1109/42.750253

[31] Cavada C (2000) Company T, Tejedor J, Cruz-Rizzolo RJ, Reinoso-Suarez F (2000) The anatomical connections of the macaque monkey orbitofrontal cortex. A review. Cereb Cortex 10: 220–242. 10731218 10.1093/cercor/10.3.220

[32] Fasold O, von Brevern M, Kuhberg M, Ploner CJ, Villringer A, et al (2002) Human vestibular cortex as identified with caloric stimulation in functional magnetic resonance imaging. Neuroimage 17: 1384–1393. 12414278 10.1006/nimg.2002.1241

[33] Wiener-Vacher SR, Mazda K (1998) Asymmetric otolith vestibulo-ocular responses in children with idiopathic scoliosis. J Pediatr 132: 1028–1032. 9627598 10.1016/s0022-3476(98)70403-2

[34] Bullmore ET, Bassett DS (2011) Brain graphs: graphical models of the human brain connectome. Annu Rev Clin Psychol 7: 113–140. 21128784 10.1146/annurev-clinpsy-040510-143934

[35] Domenech J, Tormos JM, Barrios C, Pascual-Leone A (2010) Motor cortical hyperexcitability in idiopathic scoliosis: could focal dystonia be a subclinical etiological factor? Eur Spine J 19: 223–230. 20033462 10.1007/s00586-009-1243-y PMC2899814

[36] Goldberg CJ, Dowling FE, Fogarty EE, Moore DP (1995) Adolescent idiopathic scoliosis and cerebral asymmetry. An examination of a nonspinal perceptual system. Spine (Phila Pa 1976) 20: 1685–1691. 7482018 10.1097/00007632-199508000-00007

[37] Domenech J, Garcia-Marti G, Marti-Bonmati L, Barrios C, Tormos JM, et al (2011) Abnormal activation of the motor cortical network in idiopathic scoliosis demonstrated by functional MRI. Eur Spine J 20: 1069–1078. 21499781 10.1007/s00586-011-1776-8 PMC3176702

